# Evaluation of the Application of Urea Dissociation Treatment to Reduce the False‐Positive Rate of ELISA for Syphilis Detection

**DOI:** 10.1002/jcla.70262

**Published:** 2026-06-04

**Authors:** Jiao Wang, Bin Li, Haodi Li, Xucai Dong

**Affiliations:** ^1^ Xi'an Area Medical Laboratory Center Xi'an Shaanxi People's Republic of China; ^2^ Shaanxi Lifegen Co., Ltd. Xi'an Shaanxi People's Republic of China; ^3^ Wuhan Central Hospital of the Third Bureau of CECEC Wuhan Hubei People's Republic of China

**Keywords:** ELISA, false positive rate, syphilis, urea

## Abstract

**Objective:**

Exploring the application of urea dissociation treatment to reduce the false‐positive rate of ELISA for syphilis detection.

**Methods:**

From January 2023 to January 2024, eleven false‐positive syphilis samples were collected from Xi'an Regional Medical Laboratory Centre as the experimental group, five positive specimens as the control group, and five negative specimens as the control group. All specimens were divided into three groups: A, B, and C. They were treated with 6 mol/L urea for 10 min, 6 mol/L urea for 20 min, and 10 mol/L urea for 10 min, respectively, and then processed according to the experimental procedures. Combined with the analysis of the TPPA and TP immunoblotting results, the Wilcoxon test was used to compare the S/CO values of each group.

**Results:**

Specimens 2 and 3 still had no < 1.0 S/CO in group A, while B and C had < 1.0 S/CO after completion of the separation. Meanwhile, specimens 7 and 8 of the experimental group had S/CO close to 1.0 after dissociation in all three groups. The positive control group had an S/CO ratio of greater than 1.0 in all three groups. The negative control specimens, both before and after separation, had no effect. The differences in S/CO values between experimental groups A, B, and C before and after dissociation were statistically significant (*p* < 0.05). The values of TP are below the manufacturer's allowable imprecision requirements.

**Conclusion:**

Urea dissociation by ELISA is effective in reducing the false‐positive rate of syphilis.

## Graphical abstract

Exploring the application of urea dissociation treatment to reduce the false‐positive rate of ELISA for syphilis detection. From January 2023 to January 2024, seven false‐positive syphilis samples were collected from Xi'an Regional Medical Laboratory Centre as the experimental group, five positive specimens as the control group, and five negative specimens as the control group. All specimens were divided into groups A, B, and C. They were treated with 6 mol/L urea for 10 min, 6 mol/L urea for 20 min, and 10 mol/L urea for 10 min, respectively, and then processed according to the experimental procedures. Combined with the analysis of the TPPA and TP immunoblotting results, the Wilcoxon test was used to compare the S/CO values of each group. Specimens 2 and 3 still had no < 1.0 S/CO in group A, while B and C had < 1.0 S/CO after completion of the separation; both specimens were positive for IgG antibody P45. Meanwhile, specimens 7 and 8 of the experimental group had S/CO close to 1.0 after dissociation in all three groups. The positive control group had the S/CO > 1.0 in all three groups. The negative control specimens before and after the separation had had no effect. The differences in S/CO values between experimental groups A, B, and C before and after dissociation were statistically significant (*p* < 0.05). The values of TP are below the manufacturer's allowable imprecision requirements. Urea dissociation by ELISA is effective in reducing the false‐positive rate of syphilis.

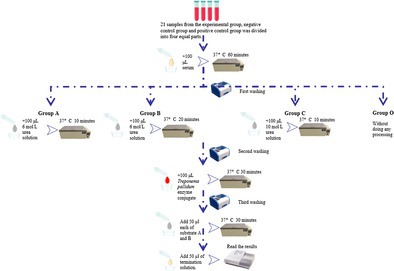

## 1 Introduction

Syphilis is an acute and chronic infectious disease caused by *Treponema pallidum* (TP). Infection with the organism can cause damage to multiple systems and organs in the human body, resulting in a variety of clinical manifestations that lead to tissue damage, dysfunction, and even life‐threatening conditions [1]. Current laboratory detection methods for syphilis include the *Treponema pallidum* test and serological testing, which is divided into non‐treponemal and treponemal tests. The most widely used non‐treponemal tests are the Venereal Disease Research Laboratory (VDRL) test and the rapid plasma reagin (RPR) test. Due to their low specificity and sensitivity, non‐treponemal tests can only assess the activity of the infection and monitor dissociation [2]. Tests for *Treponema pallidum* include electrochemiluminescence immunoassay (ECLIA), chemiluminescence immunoassay (CLIA), enzyme‐linked immunosorbent assay (ELISA), *Treponema pallidum* particle assay (TPPA), syphilis immunoblot ( *Treponema pallidum* ‐Western blot, TP‐WB) [3]. TPPA uses an antigen prepared from the attenuated strain of TP to induce an agglutination reaction with antibodies in the serum, resulting in high sensitivity and specificity. The US Centers for Disease Control and Prevention recommends TPPA as the diagnostic method [2].

Currently, the clinical diagnosis of syphilis requires careful consideration of several factors, including medical history, clinical manifestations, and laboratory test results. However, the clinical manifestations of syphilis are incredibly complex, and laboratory conditions limit the detection of the aetiological agent of syphilis. Therefore, syphilis serological testing has become the primary basis for diagnosis [2]. Compared with other serological methods, ELISA can be used for screening tests. However, this test is also susceptible to false positives due to the influence of factors such as the body's disease, specimen hemolysis, equipment, reagent quality, methodology, and human factors. Most of the current research focuses on cases of false‐positive syphilis [4, 5] or the analysis of false‐positive results [6, 7, 8]. There are relatively few reports on how to reduce false‐positive results through improved methods. Only Liu Suyan et al. [9] used calf serum to inhibit false‐positive reactions in ELISA, and Wang Qiang et al. [10] used urea dissociation to reduce false‐positive results.

This study investigated whether treating test samples with 6 mol/L urea for 10 min, 6 mol/L urea for 20 min, and 10 mol/L urea for 10 min, respectively, could reduce false positives and misdiagnoses of syphilis using the original ELISA method.

## 2 Materials and Methods

### 2.1 Objects and Materials

#### 2.1.1 Study Subjects

Seventeen serum samples collected from January 2023 to January 2024 at the Xi'an Regional Medical Laboratory Center were used in this experiment.

The inclusion criteria for the experimental group were: S/CO value of 1–4 in the ELISA test and a negative TPPA result for a total of 11 samples; the inclusion criteria for the positive control group were: S/CO value > 4 in the ELISA test and a positive TPPA result, for a total of 5 samples; the inclusion criteria for the negative control group were: S/CO value < 1 in the ELISA test and a negative TPPA result, for a total of 5 samples. The selection criteria for the experimental and control groups were determined during the initial sample collection.

#### 2.1.2 Reagents and Instruments

The kits used in the immunoblotting method were Anti‐Syphilis Spirochete and Cardiolipin Antibody IgG Detection Kit (LOT. DY2112‐1601G) and Anti‐Syphilis Spirochete and Cardiolipin Antibody IgM Detection Kit (Batch No. DY2112‐1601 M), which were both produced by OMON Medical Laboratory Diagnostics AG; the kit used in the ELISA method was the Diagnostic Kit for Syphilis Spirochete Antibody (LOT. The ELISA kit was the Syphilis Spirochete Antibody Diagnostic Kit (LOT N20230724)), produced by Beijing Wantai Bio‐pharmaceuticals Co. Syphilis spirochete antibody chemiluminescence was performed using an Alinity I chemiluminescence analyser from the Abbott Group of companies (LOT 50281BE00). TPPA reagent (LOT. WN81029), purchased from Fuji Rebio Co. Ltd.; The ELISA was performed using an enzyme labelling analyser (RT‐6100) from Shenzhen Radiology Life Science Co. Incubation was performed using a triple‐use thermostatic electric water heater (HHW‐600D) from Tianjin Tester Instrument Co. 1.2 Experimental Methods.

#### 2.1.3 ELISA Method Treating Samples With Different Methods, Respectively

**FIGURE 1:**
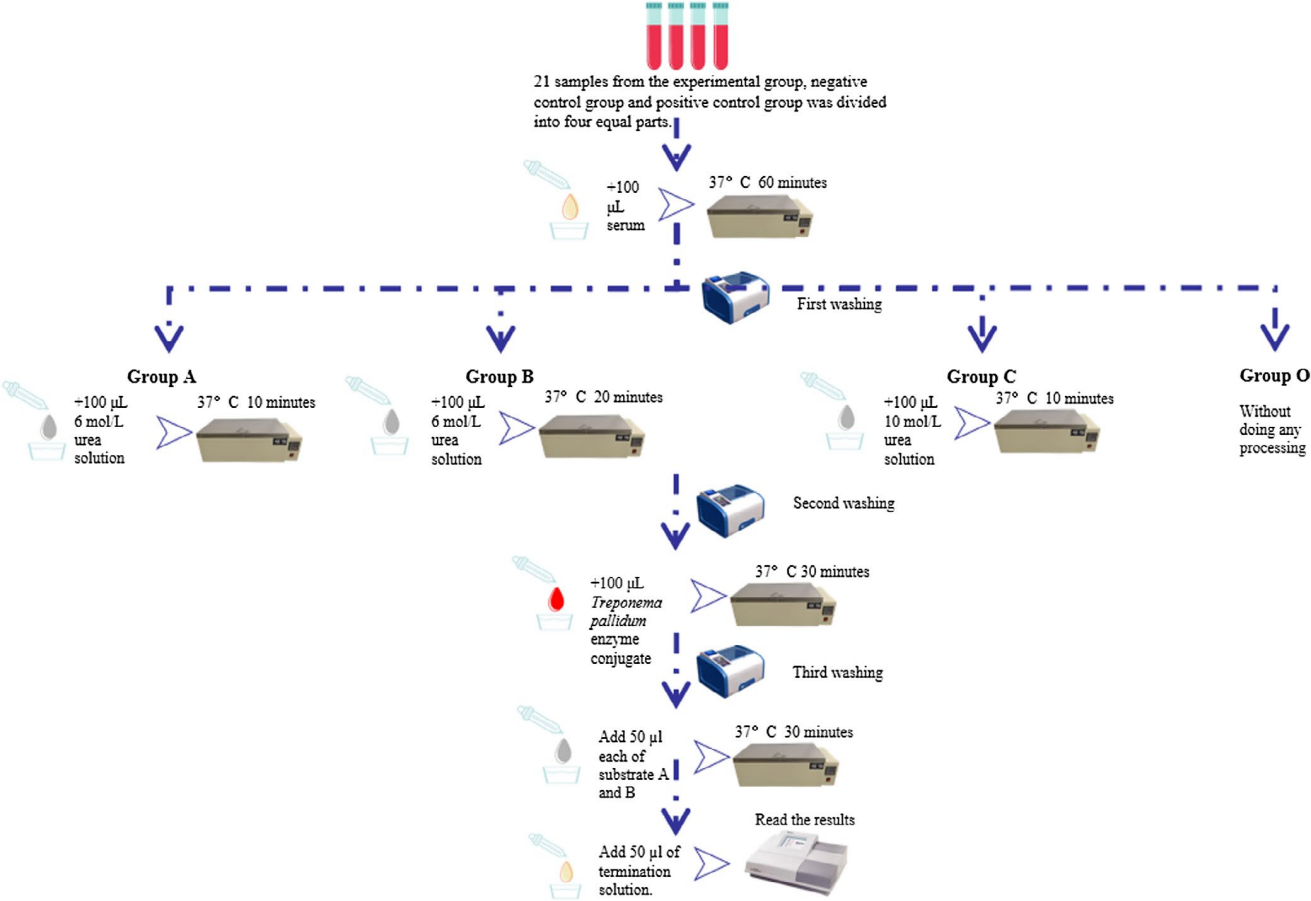
Flow chart of the ELISA experiment.

Each sample from the experimental group, negative control group, and positive control group was divided into four equal parts, following a comprehensive experimental design. Group A was treated with 6 mol/L urea for 10 min, group B with 6 mol/L urea for 20 min, group C with 10 mol/L urea for 20 min, and group O without any processing. The specific steps are shown in Figure 1. The urea treatment concentration and time selection were based on the research of Wang Qiang et al.

#### 2.1.4 Western Blotting

In this experiment, the immunoblotting (Western blotting) kit contains electrophoretically separated strips of detection membrane for the syphilis spirochete antigen. During the first step of warming, the diluted serum reacts with the detection membrane strips. If the sample is positive, the specific IgG or IgM binds to the antigen. To detect bound antibodies, an enzyme conjugate of enzyme‐labelled anti‐human IgG/IgM is added for the second step of incubation, followed by the addition of enzyme substrate to produce an observable colour reaction.

Criteria for IgG positivity: more than one coloured p15kDa, p17kDa, p45kDa, p47kDa bands; criteria for IgM positivity: at least one coloured p15kDa, p17kDa, p45kDa, p47kDa bands.

#### 2.1.5 Chemiluminescence (CL)

The 21 specimens were tested using an Abbott salinity I chemiluminescence immunoassay analyzer. The instrument was fully automated, from testing to result interpretation, and the built‐in judgment criterion of S/CO > 1.0 for anti‐TP indicated reactivity, while S/CO < 1.0 indicated non‐reactivity.

#### 2.1.6 TPPA Method

TPPA test, manual operation, according to the instructions of the reagent, visual interpretation of the results, the reaction hole in the smooth button shape is negative, and the appearance of agglutination in the form of irregular deposition is positive.

### 2.2 Methodological Review

After changing the reaction procedure, the experimental methodology was reviewed with syphilis antibody‐positive specimens.

#### 2.2.1 Precision Test

Validation was carried out in accordance with the national health industry standard, WS/WS/T492‐2016, ‘Performance Verification of Precision and Correctness for Determination of Quantitative Items in Clinical Tests’. The specific validation programme is as follows:

By the improved method, five consecutive days of determination, one analytical batch per day, two concentration levels per batch, and each concentration level was repeated three times using the same sample.

#### 2.2.2 Conformity

The experimental protocol refers to CNAS‐GL038′ Guidelines for Performance Validation of Qualitative Clinical Immunology Test Procedures' using candidate methods to assess samples for known proficiency testing or inter‐laboratory quality assessment and comparison between different methodologies, and/or the same method between different laboratories. Negative samples (including at least five samples positive for other markers), positive samples (including at least five weakly positive samples with concentrations between the cut‐off value and 2–4 times the cut‐off value, and one very high value positive), a total of 20 samples, randomly divided into groups of 4, are selected. Eligibility criteria: a negative compliance rate and a positive compliance rate of ≥ 80%.

### 2.3 Statistical Analysis

SPSS24.0 software was used for statistical analysis, the Shapiro–Wilk test was used to test the normality of the measurement data, the sample data did not meet the normal distribution, the Wilcoxon test was used to analyze the changes in the S/CO value of the experimental group after different dissociations, and the *χ* ^2^ test was used to investigate the effect of different methods on the reduction of false‐positive syphilis for the strategies that can reduce the S/CO value, with *p* < 0.05 as the value of false‐positive syphilis. The *χ* ^2^ test was used to analyze the role of different methods in reducing false‐positive syphilis. The difference was considered statistically significant at *p* < 0.05.

### 2.4 Sample Size and Power Calculation

This study employed a paired design to validate the equivalence of the original detection method (X) and the results obtained after urea dissociation treatment (Y) for detecting weakly positive samples. Primary outcome measure: the difference in S/CO values between the two groups (Δ = X‐Y). Parameters set: clinical equivalence limit δ = 0.2 S/CO (based on the CLIA'88 allowable error), standard deviation of the difference *σ* = 0.21 (derived from the pre‐experiment), α = 0.05 (two‐sided), power = 90%. The calculated minimum sample size is 38 pairs (expanded to 42 pairs to account for a 10% dropout rate). According to the current sample size, the power is calculated to be 0.81.

## 3 Results

### 3.1 Laboratory Characteristics of the Study Subjects

The experimental group's samples were positive by ELISA and negative for syphilis spirochete IgM and IgG class antibodies by immunoblotting. Five positive control samples were positive by ELISA and positive for IgG and IgM by immunoblotting. Five negative control samples were negative by ELISA and negative for IgG and IgM by immunoblotting. All specimens were tested for syphilis‐specific antibodies (CL) and TPPA. Results are shown in Table 1.

**TABLE 1:** Differences in S/CO values of the three groups after dissociation with different methods respectively.

Sequence number	Group O: (S/CO)	Group A: (S/CO)	Group B: (S/CO)	Group C: (S/CO)	Chemiluminescence (COI)	TPPA	Immunoblotting IgG					Immunoblotting IGM				
							p15	p17	p45	p47	P22	p15	p17	p45	p47	P22
Experimental group																
1	1.01	0.73	0.01	0.03	0.21	−	−	−	−	−	−	−	−	−	−	−
2	3.42	1.41	0.82	0.86	0.42	−	−	−	+	−	−	−	−	−	−	−
3	3.78	1.59	0.77	0.79	0.73	−	−	−	+	−	−	−	−	−	−	−
4	1.45	0.71	0.36	0.68	0.13	−	−	−	+	−	−	−	−	−	−	−
5	1.77	0.11	0.02	0.05	0.00	−	−	−	+	−	−	−	−	−	−	−
6	1.84	0.01	0.03	0.06	0.38	−	−	−	−	−	−	−	−	−	−	−
7	2.36	0.98	0.78	0.81	1.02	−	−	−	−	−	−	−	−	−	−	−
8	2.11	0.77	0.81	0.95	1.14	−	−	−	−	−	−	−	−	−	−	−
9	3.79	0.01	0.02	0.03	0.08	−	−	−	−	−	−	−	−	−	−	−
10	1.12	0.03	0.04	0.06	0.22	−	−	−	−	−	−	−	−	−	−	−
11	1.78	0.44	0.32	0.40	1.34	−	−	−	+	−	−	−	−	−	−	−
Positive control group																
12	4.67	4.22	3.61	3.87	3.58	+	+	+	+	−	−	−	−	+	−	−
13	4.02	2.81	1.43	2.44	2.01	+	+	+	+	−	−	−	−	−	+	−
14	10.31	8.12	8.21	8.14	21.62	+	+	−	+	+	−	+	+	+	+	−
15	5.24	2.17	1.78	1.68	3.97	+	+	−	+	−	−	−	+	+	−	−
16	6.89	2.97	2.42	2.12	5.97	+	−	−	+	−	−	+	+	+	−	−
Negative control group																
17	0.01	0.01	0.02	0.02	0.02	−	−	−	−	−	−	−	−	−	−	−
18	0.01	0.02	0.02	0.01	0.01	−	−	−	−	−	−	−	−	−	−	−
19	0.01	0.01	0.01	0.01	0.02	−	−	−	−	−	−	−	−	−	−	−
20	0.01	0.01	0.01	0.02	0.02	−	−	−	−	−	−	−	−	−	−	−
21	0.01	0.02	0.01	0.02	0.01	−	−	−	−	−	−	−	−	−	−	−

*Note:* TPPA: Treponema pallidum particle agglutination test; Group O: Without doing any processing; Group A: 6 mol/L Urea dissociation 10 min; Group B: 6 mol/L Urea dissociation 20 min; Group C: 10 mol/L Urea dissociation 10 min.

As seen in Table 1, specimens 2 and 3 still had no specimens < 1.0 S/CO in group A, while B and C had < 1.0 S/CO after completion of the separation; both specimens were positive for IgG antibody P45. Meanwhile, specimens 7 and 8 of the experimental group had S/CO close to 1.0 after dissociation in all three groups. The positive control group had an S/CO ratio greater than 1.0 in all three groups. The negative control specimens, both before and after separation, had no effect. As shown in Table 2, the differences in S/CO values between experimental groups A, B, and C before and after dissociation were statistically significant (*p* < 0.05).

**TABLE 2:** Changes in S/CO values in the false positive group after different dissociations analysed by paired *t* test.

Serial number	Group A—Group O	Group B—Group O	Group C—Group O
*Z*	5.855	6.883	6.346
*P*	0.000	0.000	0.000

*Note:* Group O: Without doing any processing; Group A: 6 mol/L Urea dissociation 10 min; Group B: 6 mol/L Urea dissociation 20 min; Group C: 10 mol/L Urea dissociation 10 min.

Table 3 shows that the mean values of the two TP level concentrations were 12.96 S/CO and 6.740 S/CO. The CVs for intra‐batch imprecision were 7.02% and 8.04%, and for inter‐batch imprecision were 6.76% and 7.44%, respectively. These values are beinfected with syphilis, detecting TP protein in the patient's serum is of great value for the diagnosis of syphilis [11]; TP outer membrane proteins are the primary targets of immunity, and related studies have identified nine immunoactive proteins, namely, TPN 15, TPN 17, TPN 33, TPN 37, TPN 39, TPN 43, TPN 45, TPN47 and TPN 97, while the antibody expression levels of TPN 15, TPN 17, TPN45 and TPN47 are considered to play an essential role in the pathogenesis and diagnosis of syphilis [12]. The Western blotting method, which combines recombinant complex antigens of TpN15, TpN17, TpN47, and TpN47, increases sensitivity and allows for the simultaneous detection of IgM and IgG type antibodies [13, 14]. TP‐ELISA is a serological test for syphilis that has gained recognition in recent years. The principle of TP‐ELISA is often based on the ‘double‐antigen sandwich method.’ This method can also be performed in large quantities due to the ability to use automated detection systems. However, this method may also increase the likelihood of cross‐reactivity between specific antigens and crossover antibodies, potentially leading to false positives [15, 16]. The low antigen concentration at the time of antigen encapsulation in ELISA for TP has the potential to cause non‐specific binding of proteins and enzyme markers, resulting in interference. Urea is a dissociative agent that can solubilise and denature proteins [17]. Therefore, we investigated whether different concentrations and times of urea dissociation could be used to reduce false‐positive results in ELISA for syphilis.

**TABLE 3:** Results of precision validation after modified ELISA.

Norm	Item TP (S/CO)	
Average value	12.96	6.740
Average variance within lot	0.827	0.294
Intra‐lot imprecision SD	0.910	0.542
Intra‐lot imprecision CV	7.02%	8.04%
Lot‐to‐lot imprecision SD	0.877	0.502
Lot‐to‐lot imprecision CV	6.76%	7.44%
Manufacturer's allowable in‐lot imprecision	15%	15%
Manufacturer's allowable lot‐to‐lot imprecision	15%	15%

In this study, thanks to Wang Qiang et al.'s research findings on urea concentration and separation time, Group A used 6 mol/L urea for a 10‐min separation treatment to reduce the S/CO value of false‐positive samples [10]. Two false‐positive specimens failed to be reduced to negative; this may be due to the limitation of Wang Qiang et al.'s study, which had a small number of specimens. Therefore, the present study was supplemented with additional experiments by increasing the urea concentration or the dissociation time. Group B was treated with 6 mol/L of urea for 20 min, and group C was treated with 10 mol/L of urea for 20 min. The difference in S/CO values between these three groups and the control group was statistically significant. Both groups, B and C, were able to correct the S/CO of specimens 2 and 3 to less than 1.0, which is inconsistent with the previous study [9]; this suggests that the more concentrated specimens require higher concentrations and more extended dissociation concentrations; the earlier study failed to cover enough data, and the present study attempted more possibilities. In the experimental group, IGG P45 immunoblotting was positive in 45.45% (5/11) of the cases. In contrast, the positive control group had more target antigens, which explains the lower S/CO values for the TP assay of specimens from the experimental group.

The principles and steps of the improved ELISA and the original ELISA were almost identical, except that the improved ELISA included an additional step to add the dissociation reagent for 10 min. We did not evaluate other properties of the enhanced ELISA and did not reset the critical values; instead, we used the critical value judgment method of the original ELISA. Using the original ELISA reaction system enables a more straightforward detection process that can be more easily applied and disseminated.

The precision verification found that these values for intra‐batch imprecision and inter‐batch imprecision were below the manufacturer's allowable imprecision requirements.

The limitations of this study are primarily reflected in two key aspects. Insufficient number of false‐positive samples: Due to the difficulty in collecting false‐positive samples for ELISA testing of *Treponema pallidum* ‐specific antibodies, only 11 cases were collected in the study (obtained by the laboratory within 1 year), resulting in a sample efficacy of only 0.81. This makes it impossible to validate its reliability in a larger sample size fully. Incomplete performance evaluation: The study only validated the precision and concordance rate of the ELISA after urea dissociation but did not assess other performance indicators (such as stability, interference, etc.).

## 4 Conclusion

In conclusion, urea dissociation reduces the S/CO values of false‐positive samples. Adding urea to enzyme‐linked immunosorbent assays can be used in clinical trials to supplement syphilis screening‐positive samples. However, the diagnosis of syphilis should always be evaluated in conjunction with laboratory serological test results, clinical symptoms, and epidemiological investigations to avoid misdiagnosis due to cross‐reactivity and missed diagnosis due to, for example, early syphilis infection. The specificity of the improved ELISA was not definitively assessed in this study because of the difficulty in collecting ELISA false‐positive samples, and only 11 cases were collected in the in‐house laboratory. Therefore, the conclusions of this study have some limitations, and more studies are needed to validate the effectiveness of the urea dissociation method in reducing false‐positive syphilis ELISA results. However, the dissociation experiments suggest that the experimental conditions derived from the screening results do not inherently lead to false‐positive results.

## Author Contributions

Jiao Wang: conceptualization, methodology, software, writing – original draft preparation, data curation. Bin Li: data curation, supervision, software, validation. Haodi Li: supervision, software, validation. Xucai Dong: visualization, investigation, writing – reviewing, editing.

## Funding

This work was supported by the Xi’an Science and Technology Program‑Key Industrial Chain Application Scenario Demonstration Project (Grant No. 24ZDCYYYCJ0017).

## Conflicts of Interest

The authors declare no conflicts of interest.

## Data Availability

The data that support the findings of this study are available from the corresponding author upon reasonable request.
